# Ultrasonic-assisted MoS_2_/GO/TiO_2_ ceramic coatings: Enhancing anti-friction performance through dual-interface optimization

**DOI:** 10.1016/j.ultsonch.2024.107180

**Published:** 2024-11-30

**Authors:** Ziwei Guo, Yongnan Chen, Nan Wang, Yiku Xu, Qinyang Zhao, Zhimin Hou, Guangrui Gao, Yan Kang, Haifei Zhan

**Affiliations:** a School of Materials Science and Engineering, Chang’an University, Xi’an 710064, PR China; b Western Titanium Industry Co., Ltd., Xi’an 710016, PR China; c Xi’an Surface Material Protection Co., Ltd., Xi’an 710018, PR China; d Western Metal Materials Co., Ltd., Xi’an 710201, PR China; e College of Civil Engineering and Architecture, Zhejiang University, Hangzhou 310058, PR China

**Keywords:** Ceramic coating, 2D materials, MoS_2_, Ultrasonic-assisted, Graphene oxide, Incoherent interface, Anti-friction

## Abstract

Ceramic coatings containing two-dimensional materials (2D materials) provide effective protection for light alloys during wear, significantly improving their anti-friction performance. MoS_2_ has proven highly effective in enhancing the anti-friction performance of ceramic coatings, particularly when synthesized via plasma electrolytic oxidation (PEO). However, dislocation pinning due to the incoherent interfaces in MoS_2_/TiO_2_ coatings tends to cause localized stress concentrations and brittle fracture, requiring effectively improve nanomechanical properties by optimizing interface design. To address these issues, this study used ultrasonic-assisted PEO to disperse graphene oxide (GO), which provided more possibility for *in-situ* synthesis MoS_2_, ultimately resulting in MoS_2_ with modified interlayer spacing. The change in interlayer spacing induced dislocation evolution at incoherent interface, leading to dual interface formation. At MoS_2_ (0.534 nm)/TiO_2_ interface: dislocation dipoles evolve to create considerable distortion, facilitating releasing shear stresses and inhibiting crack propagations. This process is followed by dislocation annihilation, keeping to stable interfacial bonding. Additionally, the others form strong dislocation pinning to obstruct dislocation slip and enhancing deformation resistance at MoS_2_ (0.227 nm)/TiO_2_ interface. The combined effects of dual interfacial enhancements resulted in a 90.0 % reduction in friction coefficients of the MoS_2_/GO/TiO_2_ coating compared to the traditional ceramic coating. This facile technique provides a new strategy to fabricate self-lubricating ceramic coatings on light alloys, while the introduction of ultrasound during PEO offers valuable guidance for applying ultrasound in the synthesis of 2D materials.

## 1 Introduction

Ceramic coatings have attracted considerable attention due to their applications in aerospace [1], machinery manufacturing [2], and other fields [3] to withstand high-temperature, wear and corrosion. Two-dimensional (2D materials) crystal structures [4], [5], [6] such as graphene [7], hexagonal boron nitride (h-BN) [8], and transition metal sulfides (TMDs, such as MoS_2_) [9] have moved to the focus on interest in ceramic coating. MoS_2_, in particular, has gained notable recognition because of its fascinating physical and chemical properties [10], [11], leading to different applications including anti-corrosive [12] and anti-friction coatings [13]. Notably, MoS_2_ can be synthesized during the plasma electrolytic oxidation (PEO), forming incoherent interfaces with large lattice mismatches that enhance the anti-friction performance of ceramic coatings [14].However, continuous dislocation pinning and plugging will trigger localized stress concentrations, leading the crack initiation and even brittle fracture [15]. Therefore, it is necessary to design an interface, which can effectively regulate the dislocation behavior and reduce the local stress concentration to enhance its nanomechanical properties.

It has been shown that the formation of dislocation dipoles and their evolution under different loads can cause severe distortion of the lattice, inducing large strain and thus stress release [16]. Currently one of the effective ways to introduce dislocation dipoles in 2D materials is to change the interlayer spacing. Wang et al. [17] induced the formation of disordered basal surfaces with abundant dislocation dipoles by prepared interlayer-extended MoS_2_ nanosheets, resulting in hydrogen evolution behavior facilitated by the cooperative regulation of interlayer spacing and interlayer dislocation. Similarly, the free radical-induced dislocation dipole network in multilayer graphene has been verified [18]. Therefore, it is expected that dislocation dipoles can be introduced into the *in-situ* synthesized MoS_2_/TiO_2_ interface to alleviate the stress concentration during wear and enhance the anti-friction performance of the ceramic coatings.

Graphene oxide (GO) is an oxygen-abundant material with high specific surface area, good chemical stability and excellent strength [19]. GO achieves the effect of changing the interlayer spacing of MoS_2_ in numerous electrochemical reactions, promoting ion transport and diffusion as well as ensuring the stability electrochemical reactions [20], [21]. Further, the design of MoS_2_ combining it with another 2D material having an excellent tribological properties of ceramic coatings is proven to be achievable [22], [23], [24]. Thus, it is quite feasible to introduce GO into PEO, which effects the *in-situ* synthesis of MoS_2_ to obtain anti-friction ceramic coatings with altered interlayer spacing of MoS_2_ and to generate MoS_2_/TiO_2_ incoherent interfaces with dislocation dipoles achieving alleviate stress concentration. However, GO is prone to aggregation and precipitation in alkaline electrolytes [25], leading to a decrease in its ability to effectively regulate PEO reactions, which negatively affects the *in-situ* synthesis of MoS_2_.

An effective way to improve the dispersion of nanoparticles in electrolytes and avoid agglomeration is to utilize ultrasonic assistance [26]. In sono-chemistry and sono-electrochemistry, ultrasound can fiercely promote the mass transfer process in chemical and electrochemical systems [27]. It not only leads to a good dispersion of nanoparticles, but also reduces the concentration gradient of the electrolyte, which is required for the *in-situ* synthesis of MoS_2_ in PEO reaction [27]. The formation process of PEO ceramic coatings involves a complex plasma mass transfer process between the electrolyte and the metal substrate [28], [29]. It was previously reported that ultrasonic-assisted PEO, utilizing turbulent, perturbation, and interfacial effects, can facilitate the rapid migration of charged particles, resulting in the accelerated the *in-situ* growth reaction of ceramic coatings [30]. Thus, we expect to successfully synthesize MoS_2_ *in-situ* within ceramic coatings by ultrasonic-assisted PEO reaction process, which reduces the concentration polarization of the electrolyte and promotes the homogeneous dispersion of GO, and thus optimizes the *in-situ* reaction of MoS_2_.

Inspired by the above, we try to demonstrate a synthesis strategy whereby ultrasonic assisted PEO well disperses GO to induce *in-situ* synthesis of MoS_2/_GO/TiO_2_ coating with different interlayer spacings of MoS_2_, resulting in incoherent interfaces with a large number of dislocation dipoles evolutions. Based on the design of the MoS_2_/GO/TiO_2_ coating, the morphology and composition were further characterized. Then the anti-friction performance of the coating was investigated, while the anti-friction mechanism was thoroughly studied. Finally, the dislocation evolution mechanism triggered by the MoS_2_ varieties with different interlayer spacing provides another idea for the design of anti-friction ceramic coatings on light alloys, while the introduction of ultrasound during the PEO in 2D materials provides enlightenments into the application of ultrasound in the synthesis of 2D materials.

## 2 Materials and methods

### 2.1 Fabrication of ceramic coatings

To fabricate the ceramic coatings, the first step involves the preparation of the Ti-6Al-4 V alloy with a rectangular shape. The surface was meticulously polished using sandpapers of grades 500#, 800#, and 1200# to maintain surface roughness approximately 0.32 ± 0.03 μm and remove any contaminants. The prepared Ti-6Al-4 V alloy was then placed in an aqueous solution with the composition given in Table 1, consisting of NaH_2_PO_4_, NaF, Na_2_MoO_4_, Na_2_S, and GO at various concentrations. GO used in this study is a single-layer obtained by centrifugation after preparation via the Hummers method [31]. During the PEO reaction, the samples were used as the anode, while a stainless-steel plate served as the cathode. The PEO power supply used a pulsed bipolar configuration with a frequency of 500 Hz and a duty cycle of 15 %, also as described in Table 1. The appearance of the arc discharge was visually inspected using a high-speed camera to record the breakdown voltage.

**Table 1:** Electrolyte compositions and processing parameters of PEO of different ceramic coatings.

Samples	NaH_2_PO_4_ (mmol/L)	NaF (mmol/L)	Na_2_MoO_4_ (mmol/L)	Na_2_S (mmol/L)	GO (g/L)	Frequency (Hz)	Duty cycle
Traditional PEO coating	133.36	190.52	0	0	0	500	15 %
MoS_2_ /TiO_2_ coating	133.36	190.52	29.14	384.41	0	500	15 %
MoS_2_/GO/TiO_2_ coating	133.36	190.52	29.14	384.41	5	500	15 %

**Fig. 1:**
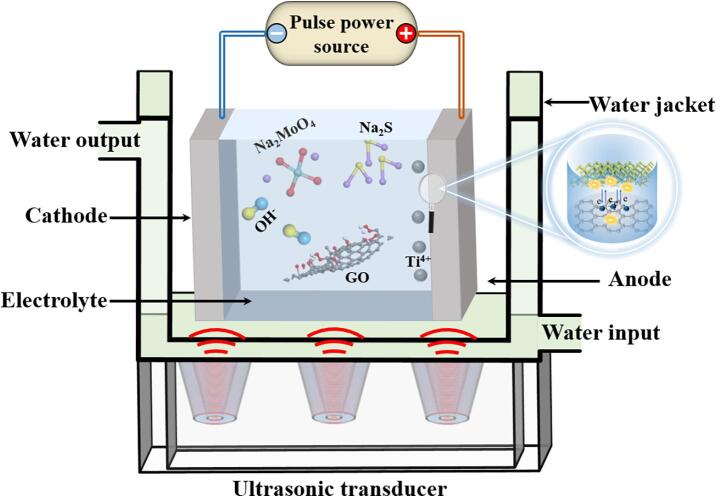
Schematic diagram of ultrasonic-assisted PEO preparation of MoS_2_/GO/TiO_2_ coating.

To enhance the synthesis process, ultrasonic assistance was applied during the PEO reaction. The ultrasonic vibrations were applied indirectly to the PEO system by connecting an ultrasonic transducer to the bottom of the cooling water sink (Fig. 1). The ultrasonic vibration unit included a sound generator with a maximum power of 100 W and a frequency of 130 kHz. The combination of ultrasonic agitation was intended to improve the dispersion of GO in the electrolyte and facilitate uniform coating formation on the alloy surface. After the preparation, we named the sample without ultrasonic assistance as ‘Non-ultrasonic MoS_2_/GO/TiO_2_ coating’ and the sample with ultrasonic assistance as ‘MoS_2_/GO/TiO_2_ coating’. The voltage and electric current were simultaneously recorded during the PEO process.

### 2.2 Characterization of MoS_2_/GO/TiO_2_ ceramic coatings

The zeta potentials of the electrolytic for MoS_2_/GO/TiO_2_ coating before and after PEO were measured by a dynamic light scattering (DLS, Malvern Zetasizer). The measurements were conducted at room temperature, with three independent measurements performed to ensure accuracy and reproducibility. Raman spectra were recorded using a Raman spectrometer (Horiba JOBIN YVONHR800), with an excitation wavelength of 532 nm and an intensity of 0.5 × 10^−5^ W. The phase composition of the MoS_2_/GO/TiO_2_ coatings was determined using X-ray diffraction (XRD) with a D/MAX-3A instrument with a Cu-Kα radiation source (λ = 1.5406 Å). The XRD analysis was conducted at an operating voltage of 40 kV and a current of 40 mA. The XRD analysis was performed with a step size of 0.02° and a scan rate of 1.5°/min over a 2θ range of 10–80°. X-ray photoelectron spectroscopy (XPS), conducted with a Thermo Scientific K-Alpha with a monochromatic Al Ka X-ray source (1486.6 eV, 6 mA and 12 kV), provided detailed insights into the chemical states and composition of the coatings. The XPS analysis was conducted at an emission angle of 60° and an analyzed area of 400 μm diameter circle, ensuring comprehensive coverage of the sample. The base pressure during the measurement was better than 2.0 × 10^−7^ Pa. Before the XPS measurement, samples were not sputter-etched for avoiding composition cluster. During spectral acquisition, the double beam neutralization was employed for charge compensation. The electronic energy analyzer operated with a pass energy of 100 eV in 1.0 eV steps for survey scans and 50 eV in 0.05 eV steps for higher resolution scans. The resulting spectral peaks were fitted using Avantage software, applying Gaussian–Lorentzian Product functions with 30 % Lorentzian and full width at half maximum (FWHM) constraints. The XPS binding energy scale was aligned by modifying the indeterminate carbon C 1 s peak. Scanning electron microscopy (SEM, Hitachi S-4800) with an energy-dispersive X-ray spectroscopy (EDS) detector (S-4800) was employed to observe the sectional and scratch microscopic features of different ceramic coatings. To ensure the interfacial state between MoS_2_ and TiO_2_, high-resolution transmission electron microscopy (HRTEM, JEM-2100F) was used at 200 kV to investigate and operate the microstructure of the nanocomposite coating. Samples were prepared using focused ion beam technology (FIB, Helios Nanolab 600i), and digital micrograph software was used to analyze the images. Fast Fourier transform (FFT) and inverse fast Fourier transform (IFFT) images were obtained to study the crystalline structures, while geometric phase analysis (GPA) maps were generated using a compatible program with Digital Micrograph and Strain++ software, providing insights into strain distribution and dislocation behavior.

### 2.3 Tribological Evaluations

The tribological behaviors of ceramic coatings were studied by means of dry friction tests, which were carried out on a pin disc friction tribometer (MMQ-02), under a ring-on-block contact configuration. The counter-face material was GCr15 ball (hardness 62–63 HRC, surface roughness 0.01 μm). The normal load is 2 N. The tests were carried out with a rotation diameter of 6 mm and rotation speed of 30 r/min. To ensure consistency and reliability of the experimental conditions, all tests were conducted in a controlled environment using an environmental chamber. The environmental chamber maintained a stable ambient temperature of 25 °C and a relative humidity of approximately 30 %. This controlled environment ensured that any variations in ambient temperature or humidity would not influence the tribological behavior of the ceramic coatings, thereby enhancing the repeatability of the experiments. All tests are performed under the guidance of the Standard Test Wear Testing with a Pin-on-Disk Apparatus (ASTM G99). The friction coefficient was recorded automatically during the test process. The sliding tests were repeated three times for reliability and reproducibility. The morphologies and depth profiles of wear tracks were observed and analyzed by means of SEM and three-dimensional surface profiler (Rtec, UP-3000) after 1200 sec test. The wear volume (mm^3^) was evaluated according to the ASTM G133-05. It can be calculated using the formula:

V=A×L

Where A is average cross-sectional area of the track (mm^2^), and L is length of the stroke (mm).

Wear rate is calculated by the following formula:

$$W=\left(\frac{V}{\left(F\times L\right)}\right)$$

Among them W is the wear rate (mm^3^/N × m), V is the wear volume (mm^3^), F was the normal load (N), and L is the sliding distance (m). A diamond probe was used for scratch test (Scratch Tester, WS-2005. China Zhongkai Co., Ltd.). The test was conducted using an electrical signal mode, with a scratch speed of 0.5 mm/s. The applied load increased linearly from 0 N to 100 N over a scratch length of 5 cm. During the test, as the probe slid across the surface of the ceramic coatings under linear loading, the electric circuit was triggered once the coating was scratched and the substrate was exposed. At this point, the collected electrical signal exhibited a change, indicating coating failure. The load at which the electrical signal was captured was defined as the critical load, serving as a criterion for evaluating the adhesion properties of the ceramic coatings. To ensure the accuracy and reliability of the experiment, the scratch test was repeated three times for each specimen.

## 3 Results

### 3.1 Ultrasound-assisted in-situ reaction of MoS_2_/GO/TiO_2_ coating

**Fig. 2:**
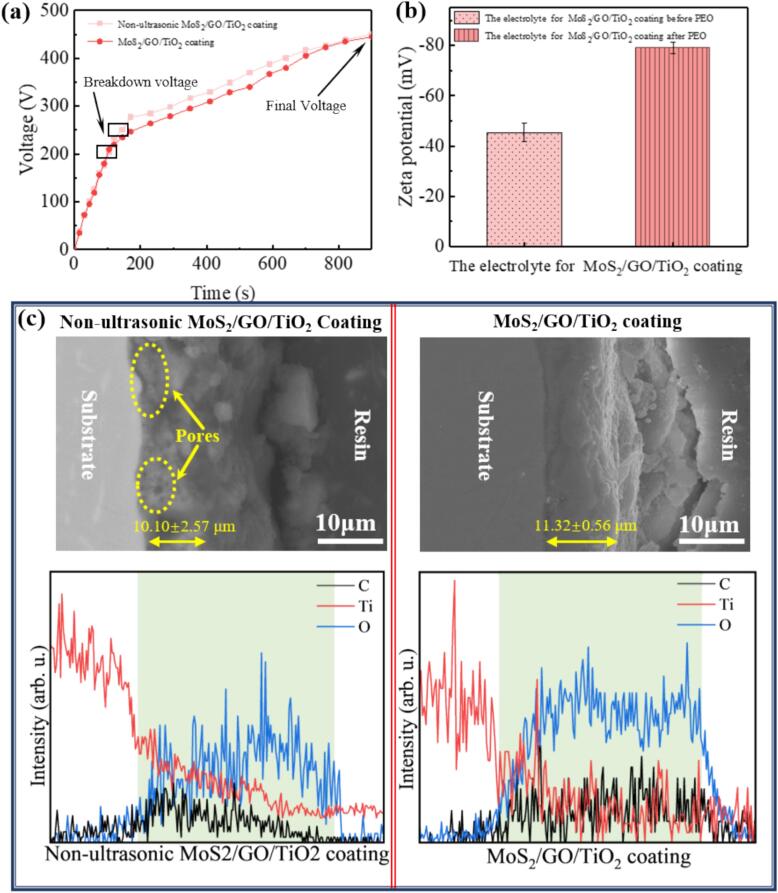
(a) Voltage-time responses for the PEO process of the Non-ultrasonic MoS_2_/GO/TiO_2_ coating and the MoS_2_/GO/TiO_2_ coating. (b) Zeta potential of the electrolyte before and after ultrasonic treatment for the MoS_2_/GO/TiO_2_ coating. (c) Cross-sectional morphologies and elemental line scans of the Non-ultrasonic MoS_2_/GO/TiO_2_ coating and the ultrasonic-assisted MoS_2_/GO/TiO_2_ coating.

In the ultrasound-assisted PEO reaction, numerous microbubbles are generated in the electrolyte due to the cavitation effect induced by ultrasonic vibrations [32]. The continuous formation and collapse of microbubbles release substantial energy, resulting in localized heating at the electrolyte/substrate interface. This heating can lead to initial defects in the dielectric layer, which subsequently become weak points that facilitate penetration at lower voltages, triggering micro-arc discharges [33]. Notably, the breakdown voltage of the MoS_2_/GO/TiO_2_ coating is reduced to 210 V, representing a 16.3 % decrease compared to the Non-ultrasonic MoS_2_/GO/TiO_2_ Coating (Fig. 2a). Ultrasonic assistance not only reduces the breakdown voltage of the PEO reaction, enabling the micro-arc discharge to occur as early as possible, but also promotes the mass transfer process within the electrolyte through ultrasound-induced mechanical effects, such as turbulence and perturbation [29], [34]. This improvement allows for a more uniform dispersion of GO in the solution, preventing agglomeration and reducing concentration polarization in the electrolyte. It can be evidenced by the zeta potential of the electrolyte after sonication, which increases by 74.2 % in the absolute value (Fig. 2b), indicating effective dispersion and preventing re-agglomeration of GO due to energy mismatch [35]. The enhanced dispersion of GO significantly increases the electron mobility rate, thereby facilitating the chemical reactions during the PEO process. The *in-situ* reaction process of MoS_2_ can be simplified [36]:

(1) $$MoO_{4}^{2-}+4S^{2-}+12H^{+}+2e^{-}\to MoS_{2}+2H_{2}S↑+{4H}_{2}O$$

In this process, the hydroxyl group (–OH) on the surface of GO can form hydrogen bonding with S^2−^ as indicated by the interaction $GO-OH\cdots S^{2-}$[37]. Under the action of hydrogen bonding, S^2−^ is more uniformly distributed under the band following of GO to enhance the collision chance between reactants. Simultaneously, ${{\text{MoO}}_{\text{4}}}^{\text{2-}}$ can form coordination bonds with carboxyl groups (–COOH) on the surface of GO, as represented by the following equation [38]:

(2) $$GO-COO^{-}+{{MoO}_{4}}^{2-}\to GO-COO-M_{O}O_{4}+3e^{-}$$

This stable coordination complex stabilizes the ${{\text{MoO}}_{\text{4}}}^{\text{2-}}$ to react with the well-dispersed S^2−^ to form MoS_2_. Thus, the uniform dispersion of GO not only enhances the interaction between S^2−^ and ${{\text{MoO}}_{\text{4}}}^{\text{2-}}$ to promote the *in-situ* formation of MoS_2_ but also ensures its even distribution throughout the ceramic coating. Furthermore, the homogeneously dispersed GO becomes polarized under a strong electric field and arranged in order according to the direction of the electric field [39], which enhances the discharge intensity of the PEO reaction and obtains denser and more uniform discharges in the MoS_2_/GO/TiO_2_ coating. The improved uniformity decreases the energy density of individual micro-discharge, leading to less molten oxide ejection. During the compression cycle of ultrasonic vibration, the small amount of molten oxide flows back into the discharge channels, filling them up and further densifying the coating, ultimately achieving lower porosity (Fig. 2c) [40]. The thickness of Non-ultrasonic MoS_2_/GO/TiO_2_ coating and the MoS_2_/GO/TiO_2_ coating is 10.10 ± 2.57 μm and 11.32 ± 0.56 μm, respectively (Fig. 2c). Consequently, the ultrasonic-assisted PEO reaction can effectively prepare a uniform and denser MoS_2_/GO/TiO_2_ coating evidenced by elemental line scans, establishing a solid foundation for the anti-friction properties of the coating (Fig. 2c).

### 3.2 Chemical composition of MoS_2_/GO/TiO_2_ coatings

**Fig. 3:**
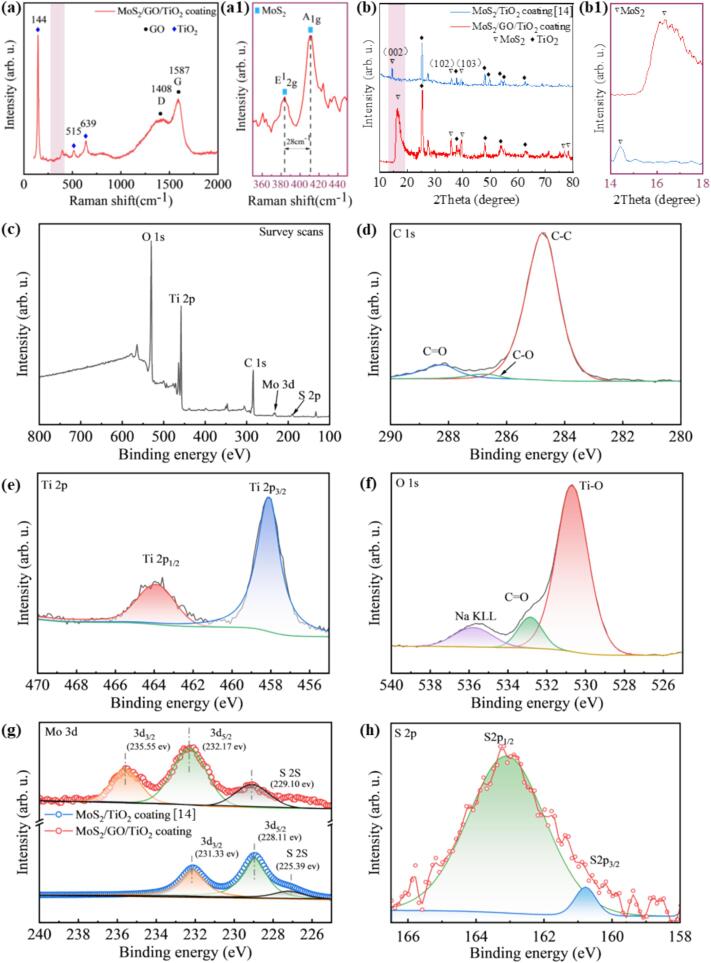
(a) Raman spectra of MoS_2_/GO/TiO_2_ coatings (The characteristic peaks located at 1480 cm^−1^ and 1587 cm^−1^ correspond to D and G peak of GO, respectively [41]). (a1) Enlarged view of the Raman spectra showing detailed peak positions of MoS_2_ (In which two peaks at 385 cm^−1^and 410 cm^−1^ could be ascribed to E^1^_2g_ and A_1g_ peak of MoS_2_, respectively [42]). (b) XRD pattern and corresponding narrow and slow sweep of XRD of different ceramic coatings. (b1) Enlarged view of the narrow XRD sweep highlighting specific diffraction peaks. (c) XPS survey spectra of MoS_2_/GO/TiO_2_ coating. High-resolution XPS spectra for: (d) Ti 2p, (e) C 1 s, (f) O 1 s, (g) Mo 3d and (h) S 2p.

It has previously reported a synthesis method for MoS_2_ in ceramic coating by *in-situ* reaction assisted by PEO. Therefore, to boost the *in-situ* synthesis reaction of MoS_2_, GO with excellent electrical conductivity was introduced to modulate the electrochemical reaction to obtain the MoS_2_/GO/TiO_2_ coating. This was evidenced by the Raman spectra (Fig. 3a), where two characteristic peaks were observed at approximately 1480 cm^−1^ and 1587 cm^−1^, which were designated as the peaks of D and G of GO, respectively [41]. Additionally, prominent peaks at approximately 385 cm^−1^and 410 cm^−1^ were identified, which can be attributed to E^1^_2g_ associated with in-plane vibrations and A_1g_ peak associated with out-of-plane vibrations of MoS_2_ (Fig. 3a1) [42]. The XRD analysis proves the presence of *in-situ* MoS_2_ and TiO_2_ which are crucial components of the coating [14]. Interestingly, two distinct peaks were observed at 35.87° and 39.53°, corresponding to the (102) and (103) planes of 2H-MoS_2_ (JCPDS No. 37e1492) (Fig. 3b) [43]. Additionally, the sharp and standard peak of the (002) plane corresponding to MoS_2_ in the MoS_2_/GO/TiO_2_ coating is stronger and shifts to a larger angle of 16.5° from 14.39° (Fig. 3b1). The stronger of the intensity confirms the effectiveness of GO to improve the synthesizing of MoS_2_ layers along (002) plane, predicting the change in the interlayer spacing. The dominant peaks presented in the survey spectra (as displayed in Fig. 3c-h were assigned as Ti 2p, C 1 s, O 1 s, Mo 3d and S 2p), implying that the MoS_2_/GO/TiO_2_ coating were primarily composed of Ti, C, O, Mo and S. The survey spectrum (as shown in Fig. 3d and f) of the GO demonstrated that the major peaks were assigned to O 1 s and C 1 s, while the high-resolution C 1 s spectral peaks at 284.8, 286.8 and 288.2 eV (Fig. 3d) were assigned to C–C, C-O and C = O bonding, respectively [44]. The band structure and local coordination characteristics as evidenced in the XPS spectra show alterations in the binding energy of MoS_2_. Specifically, the Mo 3d spectra in Fig. 3g and h indicated a slight increase in binding energy for Mo_5/2_, rising from 228.11 to 232.17 eV, and a more significant increase for S 2S, from 225.39 eV to 229.10 eV [45]. These shifts reflect modifications in the band structure and the electron levels of MoS_2_, as well as changes in the interlayer binding energies, which may be related to the participation of GO in the *in-situ* synthesis reaction of MoS_2_. The differences in the chemical composition of the ceramic coatings indicate that the *in-situ* MoS_2_/GO/TiO_2_ coating was successfully prepared. Moreover, the interlayer structure of MoS_2_ and its binding behavior were altered by GO.

### 3.3 Interlayer spacing of MoS_2_/GO/TiO_2_ coatings

**Fig. 4:**
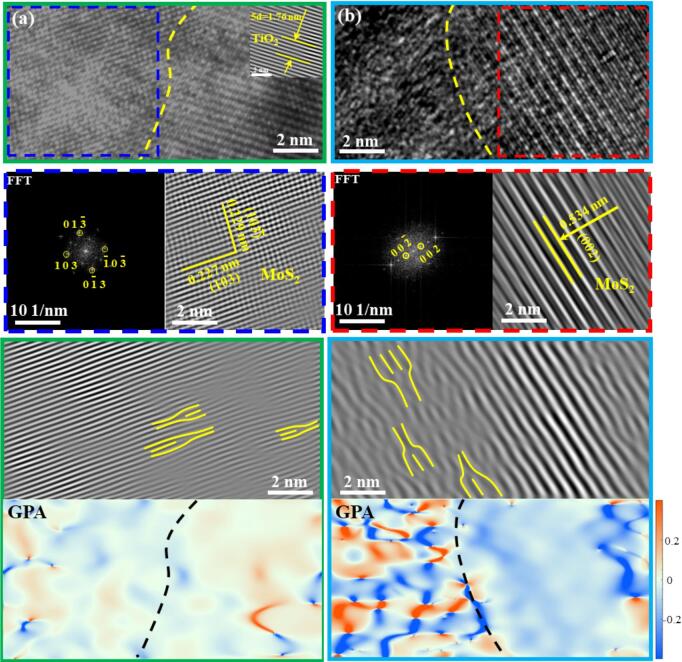
HRTEM images, Fast Fourier transform (FFT) images and corresponding Geometric Phase Analysis (GPA) mapping calculated by Digital Micrograph of interface between MoS_2_ with different interlayer spacing and TiO_2_ in the MoS_2_/GO/TiO_2_ coating: (a) MoS_2_ (0.227 nm)/TiO_2_ interface, (b) MoS_2_ (0.534 nm)/TiO_2_ interface.

Aiming to understand changes in nanostructure of MoS_2_ combined with the discussion of the chemical composition, the crystal structure and orientation of MoS_2_/GO/TiO_2_ coating were analyzed using TEM (Fig. 4). The lattice fringes of about 0.352 nm were observed (Fig. 4a), corresponding to the (1 0 1) crystal plane of TiO_2_, as confirmed by the XRD pattern [14]. HRTEM images in Fig. 4 also reveal two distinct lattice planes in the MoS_2_/GO/TiO_2_ coating, with d-spacings of 0.534 nm and 0.227 nm, respectively, corresponding to the (002) and (103) planes of 2H-phase MoS_2_ [43]. What is highly consistent with the calculated interplanar spacing based on XRD results (Fig. 3b) is that the observed spacing of (002), which can be computed as 0.534 nm by Bragg equation (Eq. (3)) [46], narrower than that of original MoS_2_ (0.615 nm).

(3) 2dsinθ=nλ

Where d is interplanar spacing, θ is diffraction angle, λ is wavelength, and n is diffraction series. This is a more intuitive demonstration of the change in the interlayer spacing of the MoS_2_ affected by GO, leading to a relatively large lattice mismatch between MoS_2_ and TiO_2_, which is categorized to the incoherent interface [15]. Based on the magnified observation (Fig. 4) and a geometric phase analysis (GPA) mapping, a small number of edge dislocations exist at the interface formed by MoS_2_ (0.227 nm) with TiO_2_ due to strain or stress incompatibility caused by thermal stresses during the PEO reaction. However, there is no stacking of dislocation and dislocations lines (Fig. 4a). However, compared to the MoS_2_ (0.227 nm)/TiO_2_ interface, there are many dislocations and distortions at the MoS_2_ (0.534 nm)/TiO_2_ interface, which demonstrates a higher susceptibility to edge dislocations and is subject to more pronounced strain, as illustrated in Fig. 4b [47]. This also indicates that dislocation density in the region of MoS_2_ (0.534 nm)/TiO_2_ interface was significantly higher than that of with MoS_2_ (0.227 nm)/TiO_2_ interface. As a result, the slip, evolution and reaction of existing edge dislocations at different interfaces (MoS_2_ (0.534 nm)/TiO_2_ or MoS_2_ (0.227 nm)/TiO_2_) will be different under shear stress, which also highlights the complex interplay between structural changes and mechanical properties, emphasizing the critical role of interfacial interactions in determining the anti-friction performance of ceramic coatings [48].

### 3.4 Anti-friction performance

**Fig. 5:**
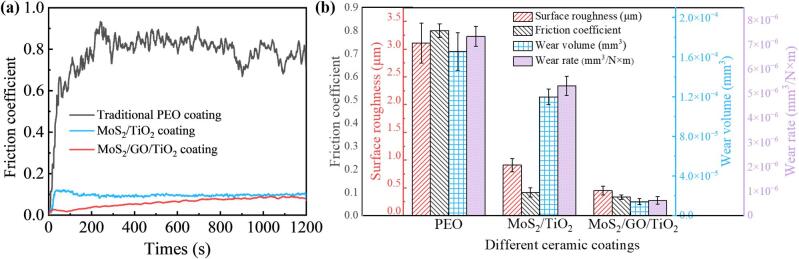
(a) The relationship between friction coefficient and time of different ceramic coatings. (b) The surface roughness, friction coefficient, wear volume and wear rate of different ceramic coatings.

In order to elucidate the tribological characteristics of MoS_2_ ceramic coating with GO addition, an analytical exploration was conducted, focusing on the surface roughness, friction coefficient, wear volume and wear rate across traditional ceramic coating, MoS_2_/TiO_2_ coating and MoS_2_/GO/TiO_2_ coating have been provided (Fig. 5). For the traditional ceramic coating, a point contact occurred and accompanied by the increase of stress concentration between the coatings and grinding pair at the beginning, causing the sharp increase of friction coefficient to a relatively value about 0.9 (Fig. 5a). With the extension of friction time, the contact interface developed into a stable wear stage. The micro-convex body was plowed. Consequently, the friction coefficient of the traditional ceramic coating stabilized within 0.8 (Fig. 5a). However, the MoS_2_/TiO_2_ coating exhibits a marked reduction in the initial friction coefficient, which decreases to 0.1–0.2 and stabilizes at 0.1 over time, representing an 87.5 % reduction compared to the traditional ceramic coating (Fig. 5a and b). Similarly, the MoS_2_/GO/TiO_2_ achieved an even lower friction coefficient of 0.08 and maintains this low value (Fig. 5a), which may be attributed to the role of GO optimizing the *in-situ* reaction process of MoS_2_, improving the overall resistance to the normal and shear stress under wear. A comparison of surface roughness, friction coefficient, wear volume and wear rate of different ceramic coatings is shown in Fig. 5b. The MoS_2_/GO/TiO_2_ coatings showed the surface roughness (0.45 μm), average coefficient of friction (0.08), wear volume (1.40 × 10^−5^ mm^3^), and wear rate (6.19 × 10^−7^ mm^3^/N × m), lower 85.5 %, 90.0 %, 91.6 % and 91.6 % than traditional ceramic coatings, which suggests that the incorporation of GO significantly improved the anti-friction performance of the MoS_2_/GO/TiO_2_ coatings.

**Fig. 6:**
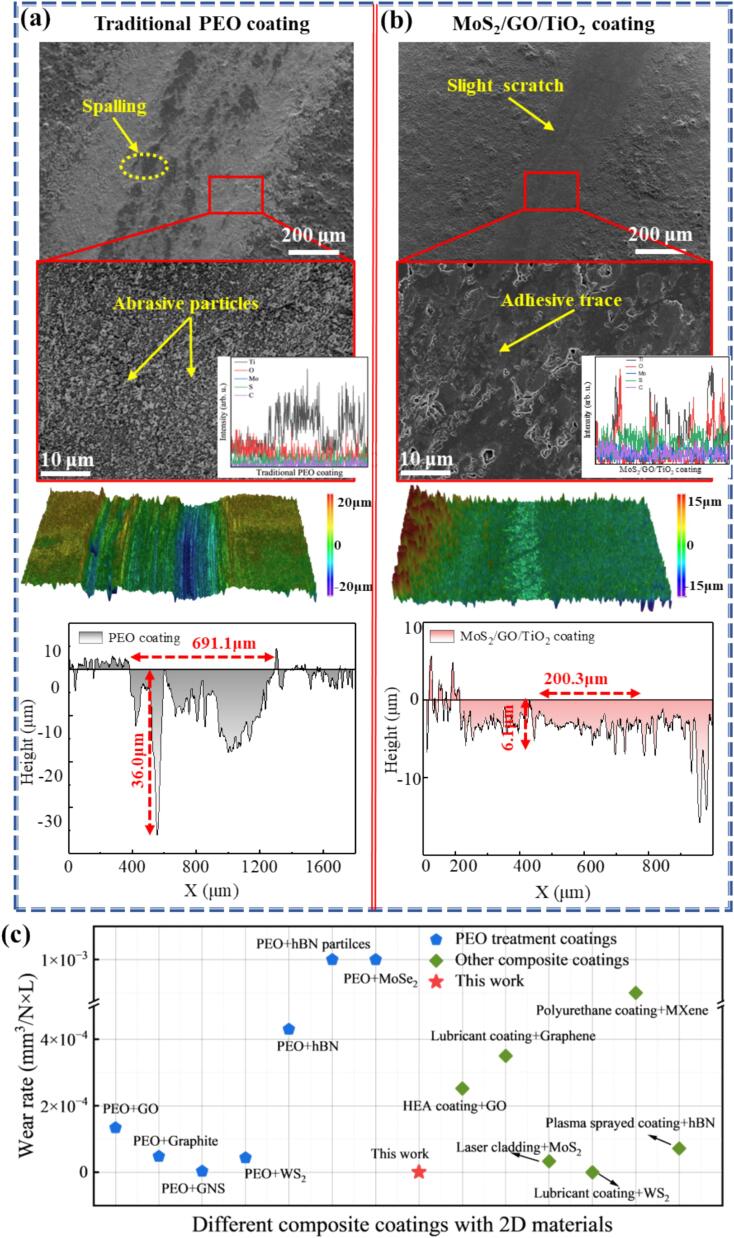
Surface morphology, three-dimensional profile shape, and two-dimensional cross-sectional profile curves and element line scans of wear tracks: (a) Traditional PEO coatings, (b) MoS_2_/GO/TiO_2_ coatings. (c) Comparison of the wear rate between this work with different composite coatings with 2D materials [44], [51], [52], [53], [54], [55], [56], [57], [58], [59], [60], [61].

After the tribological tests, the wear tracks on surface of coatings were successively investigated to evaluate the anti-friction effects of different ceramic coatings. The wear surface of the traditional ceramic coating is characterized by a relatively rough texture, exhibiting significant abrasive particles and spalling, as shown in Fig. 6. This arises from the inability of the traditional ceramic coating to form effective lubrication with the grinding pair, leading to severe abrasive wear. The presence of numerous pores, which are known to deteriorate the mechanical properties of materials [49], facilitates the detachment of hard bumps from the surface, resulting in the formation of many abrasive particles (Fig. 6a). During the wear process, these abrasive particles are pressed into the coating surface under the normal stress, while under shear stress, they move forward, creating a ploughing effect that leads to the detachment of the ceramic coating exposing the base metal, as evidenced in EDS (Fig. 6a). This interaction results in the formation of wider and deeper abrasion tracks (Fig. 6a). Notably, the furrow reaches a depth of up to 36 μm, with the primary mode of friction identified as plough wear (Fig. 6a) [50]. In contrast, the MoS_2_/GO/TiO_2_ coating reveals a much smoother worn surface, with pear groove marks that are extremely shallow, as supported by the absence of base metal exposure and the uniform distribution of elements within the coating (Fig. 6b). The width and depth of the wear tracks are reduced by 71.10 % and 83.1 % (Fig. 6b), respectively, compared with that of the traditional ceramic coating. Furthermore, the wear rate obtained in this paper are compared with that of 2D materials (graphene, GO, graphene nanosheets, WS_2_, MoSe_2_, hBN and MXene) in PEO coating and other composite coatings. The results show that the wear rate of the MoS_2_/GO/TiO_2_ coating is reduced by 82.9 % to 99.9 % compared to PEO coatings, and by 98.1 % to 99.9 % when compared to almost other composite coatings (Fig. 6c) [44], [51], [52], [53], [54], [55], [56], [57], [58], [59], [60], [61].

## 4 Discussion

### 4.1 Dislocation configurations of MoS_2_/GO/TiO_2_ coatings after wear

**Fig. 7:**
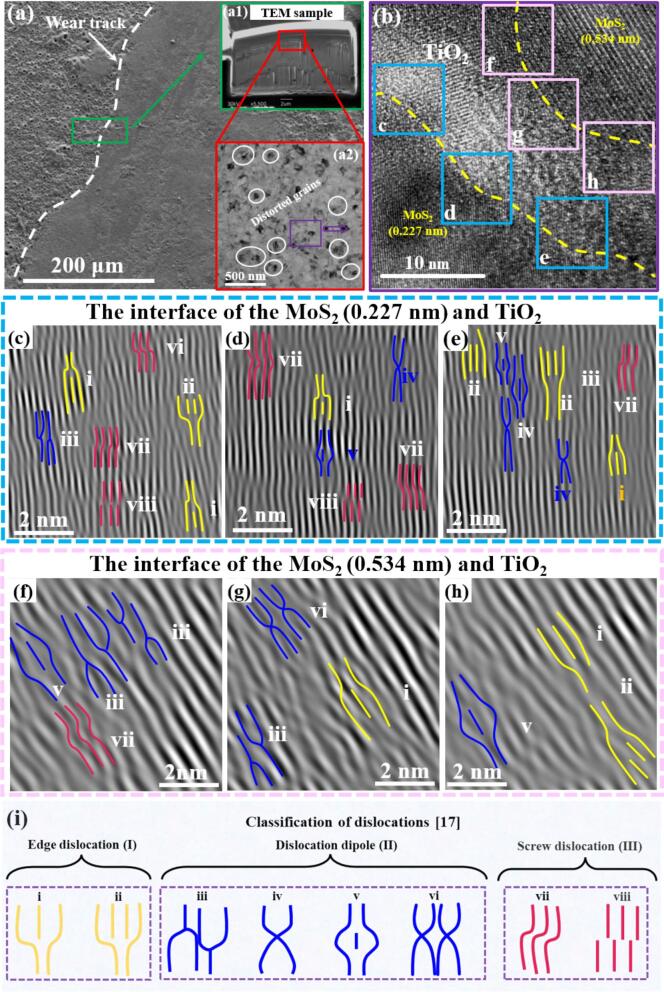
(a) Wear track morphology, corresponding image of (a1) Focused ion beam (FIB) process and (a2) HRTEM image of the MoS_2_/GO/TiO_2_ coating. (b) HRTEM image of the locally enlarged area of a2 in Fig. 7a. Interlayer dislocations sources, schematics, and HRTEM images of the interface between the different interlayer spacing MoS_2_ and TiO_2_: (c) (d) and (e) MoS_2_ (0.227 nm)/TiO_2_ interface; (f) (g) and (h) MoS_2_ (0.534 nm)/TiO_2_ interface. (i) Dislocation configurations schematic and classification containing edge dislocation, dislocation dipoles and screw dislocations [17].

In an endeavor to elucidate the high-performance anti-friction mechanism of the MoS_2_/GO/TiO_2_ Coating, FIB sampling was performed at the interface between the wear track and the unworn region, followed by high-resolution TEM analysis (Fig. 7a). From Fig. 7a1 and a2, significant grain distortion was observed, directly associated with the stress loading during the wear. Further, the typical distortion region in Fig. 7a2 was analyzed in HRTEM images, shown in Fig. 7b. Two incoherent MoS_2_/TiO_2_ interfaces with significant differences were observed, corresponding to MoS_2_ (0.227 nm)/TiO_2_ interface (highlighting with a blue box) and MoS_2_ (0.534 nm)/TiO_2_ interface (highlighting with a pink box) in Fig. 7b. At these two interfaces, various dislocation configurations were identified (Fig. 7c-h), which are geometrically line defects and can be seen as the dividing line between the sliding and non-sliding parts of the crystal [17]. Dislocations were systematically categorized into three distinct configuration: edge dislocations (Ⅰ), dislocation dipoles (Ⅱ), and screw dislocations (Ⅲ) [62]. Specifically, configurations i and ii were identified as edge dislocations, and the interlayer dislocation ii structure is formed when the free radical S atom existed at the edge of the semi-atomic plane in interlayer dislocations i and unites with the distorted parallel atomic layer (Fig. 7i). Within the dislocation dipoles (II), four distinct configurations (iii-vi) were observed, evolving from the edge dislocations (i, ii) during the wear process (Fig. 7i). Additionally, vii-viii represent screw dislocations, corresponding to relatively homogeneous strain regions (Fig. 7i) [17]. Statistical results from Fig. 7e-h show that these dislocations are coexisting in both MoS_2_ (0.227 nm)/TiO_2_ interface and MoS_2_ (0.534 nm)/TiO_2_ interface, but their distributions differ between the two interfaces, indicating that they evolve differently during the wear process.

**Fig. 8:**
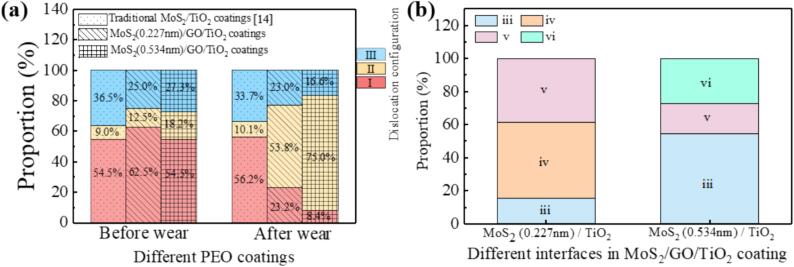
(a) Proportion of edge dislocation, dislocation dipoles and screw dislocations before and after wear of different ceramic coatings. (b) Proportion of various types of dislocation dipoles (iii-vi) in the MoS_2_ (0.227 nm)/TiO_2_ interface and the MoS_2_ (0.534 nm)/TiO_2_ interface of MoS_2_/GO/TiO_2_ coatings.

Compared to the dislocation configurations before wear (Fig. 4), both interfaces in MoS_2_/GO/TiO_2_ coating after wear exhibit not only traditional edge dislocations and screw dislocations, but also a variety of dislocation dipoles generated under combined compressive and shear stresses (as depicted in Fig. 7c-h). Statistical analysis of the dislocation configurations before and after wear was conducted, and it was found that the percentage of various configurations of dislocations hardly changes significantly before and after wear of traditional MoS_2_/TiO_2_ coating (Fig. 8a). However, comparing the dislocations configurations before and after wear of MoS_2_/GO/TiO_2_ coating, it must be pointed out the percentage of dislocation dipoles increases dramatically regardless on both incoherent interfaces (Fig. 8a). The occupation of dislocation dipoles at the MoS_2_ (0.227 nm)/TiO_2_ and MoS_2_ (0.534 nm)/TiO_2_ interfaces increased by 76.77 % and 75.73 % than those before wear, respectively (Fig. 8a). It can be inferred that these dislocation dipoles are formed through the interaction of pre-existing edge dislocations with stress before wear sliding, indicating that intricate dislocation evolution and reactions occur at the interfaces during the wear. Furthermore, the specifics of dislocation evolution at both two interfaces, however, are quite different. Dislocation dipole iv and v are dominate the MoS_2_ (0.227 nm)/TiO_2_ interface, as shown in Fig. 8b. Simultaneously, at the MoS_2_ (0.534 nm)/TiO_2_ interface, the dislocation dipoles are mainly concentrated in configurations iii and vi (Fig. 8b). The reason for this difference may be that GO induces the change in the interlayer spacing of the MoS_2_, which alters the bonding of the MoS_2_ (0.534 nm)/TiO_2_ interface incoherent interface, and thus affects the process of dislocation generation, accumulation and annihilation. Consequently, the configuration and distribution of dislocations at different interfaces highlight the complex interactions between different types of dislocations in the wear process, which will directly affect the coordination and reinforcement mechanism between the interfaces.

### 4.2 Dual-interface enhancement mechanism of MoS_2_/GO/TiO_2_ ceramic coatings

**Fig. 9:**
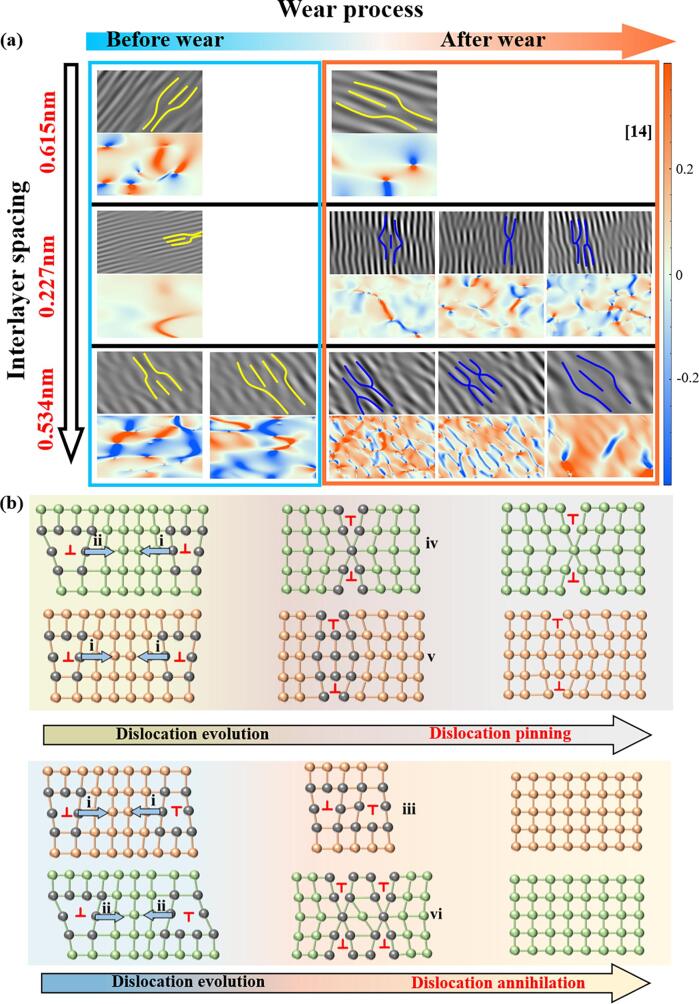
(a) Dislocation configurations and corresponding GPA mappings of MoS_2_/TiO_2_ coatings and MoS_2_/GO/TiO_2_ coatings with different MoS_2_ interlayer spacings before and after wear. (b) Schematic diagram for the evolution of different types of dislocation dipoles under stress.

The evolution of interfacial mismatch dislocations and the formation of stacking faults improves weak interactions between incoherent interfaces with large mismatches due to poorly aligned atoms as it accommodates lattice mismatches and releases stress [63], [64]. In the traditional MoS_2_/TiO_2_ coating, there is single edge dislocations before and after wear, and almost no evolution still maintains the state of weak interaction (Fig. 9a). In contrast, the MoS_2_/GO/TiO_2_ coating exhibits changes in the interlayer spacing (as evidenced by XRD pattern), resulting in a larger mismatch, which in turn promotes dislocation evolution during the wear process (Fig. 7). Specifically, when two dislocation I are near each other, they are easily attracted and form dislocation dipoles II, as illustrated in the schematic diagram in Fig. 9b. This will enhance strong interface interaction for improving the comprehensive performance of ceramic coatings (Fig. 9a b)[16]. The dislocation evolution differs significantly between the two incoherent interfaces observed in the MoS_2_/GO/TiO_2_ coating. At the MoS_2_ (0.227 nm)/TiO_2_ interface, the most are one i dislocation and one ii dislocation, two i dislocation, attract and close to each other, evolved into iv and v dislocation dipoles (Fig. 9b) [16], respectively, which proportion of iv and v dislocation dipoles after wear is as high 46.16 % and 38.46 %, respectively (Fig. 8b). At the MoS_2_ (0.534 nm)/TiO_2_ interface, the most occurring dislocation evolutions involve pairs of opposite Burgers vector dislocations i and ii evolving into dislocation iii and vi (Fig. 9b). After wear, the proportions of iii and vi dislocations dipoles is as high as 54.55 % and 27.27 %, respectively (Fig. 8b) [16]. The evolution of dislocations extensively occurs at various interfaces within the MoS_2_/GO/TiO_2_ coating, and the resulting dislocation mismatch network inevitably leads to strong interactions at incoherent interfaces [65]. However, different forms of dislocation evolution have varying effects and mechanisms on interface enhancement.

**Fig. 10:**
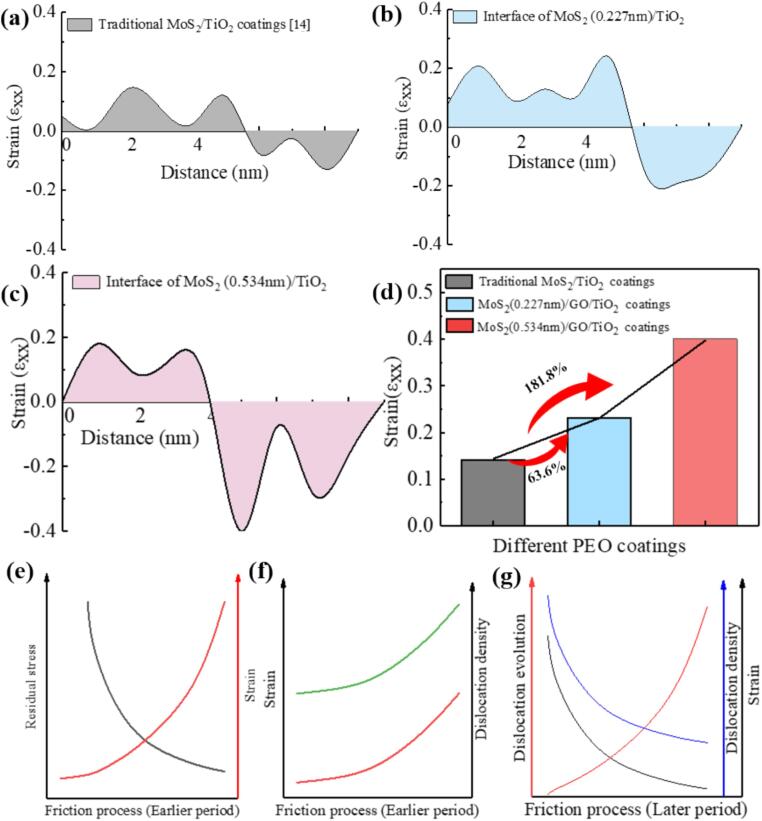
Strain analysis different ceramic coatings after wear: (a) Traditional MoS_2_ /TiO_2_ coatings, (b) MoS_2_(0.227 nm)/TiO_2_ interface in MoS_2_/GO/TiO_2_ coatings, and (c) MoS_2_(0.534 nm)/ TiO_2_ interface in MoS_2_/GO/TiO_2_ coatings. (d) Statistics of maximum strain value after wear of different ceramic coatings. (e) Trend plot of residual stress and strain during wear in MoS_2_/GO/TiO_2_ coatings. (f) Trend plot of strain and dislocation density in MoS_2_/GO/TiO_2_ coatings. (g) Trend plot of dislocation evolution, dislocation density and strain in MoS_2_/GO/TiO_2_ coatings.

**Fig. 11:**
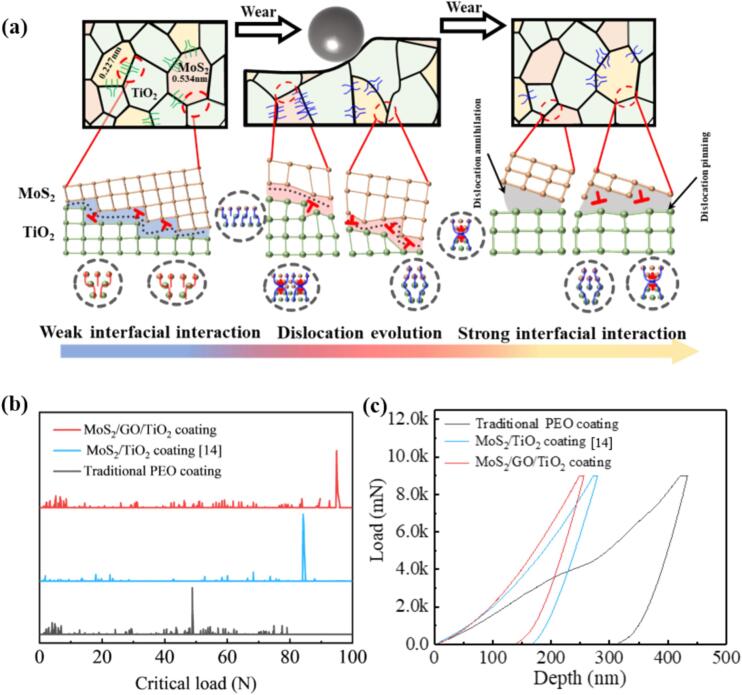
(a) Schematic diagram of the dislocation evolution and strain variation at MoS_2_ (0.227 nm)/TiO_2_ interface and MoS_2_ (0.534 nm)/TiO_2_ interface in MoS_2_/GO/TiO_2_ coatings during wear. (b) Critical load obtained by scratch tests of different ceramic coatings. (c) Load-displacement curves from nanoindentation tests of different ceramic coatings.

The strong interactions at the interfaces vary depending on the dislocation evolution occurring at each specific interface. This variability is particularly evident at the MoS_2_ (0.534 nm)/TiO_2_ interface, where the evolution process of dislocation dipoles (iii and vi) under shear stress leads to the gradual formation of distortion regions around the dislocations (Fig. 7f-h). These regions, being in close proximity, gradually merged, resulting in a 181.8 % higher strain compared to the traditional MoS_2_/TiO_2_ coating (Fig. 10a, c, d, e, and f). The increased strain arises from the conversion of shear stress into strain energy stored within the dislocations, which also facilitates the release of shear stresses [16]. This process alleviates stress concentration, preventing the formation and propagation of cracks, ultimately maintaining the protection of the substrate alloy near the interface (Fig. 10g). As a result, the critical load in the Fig. 11b of the MoS_2_/GO/TiO_2_ coating was improved by 93.8 % and 11.2 % compared to traditional ceramic coating and the MoS_2_/TiO_2_ coating, respectively. Over time, the application of shear stress causes the iii and vi dislocation dipoles to undergo a collinear reaction (Fig. 9b), eventually leading to dislocation annihilation (Fig. 11a) [16], [65]. The annihilation of these dislocations releases the strain energy previously stored within them, thereby maintaining the order and stability of the interface [66].

The evolution towards iv and v dislocation dipoles, which are relatively stable structures, occurs at the MoS_2_ (0.227 nm)/TiO_2_ interface (Fig. 9). During subsequent wear, these dislocation dipoles become entangled and a continuous dislocation pinning is found at the interface, which produces a reinforcement effect and hinders the slip of edge dislocations (Fig. 10b, Fig. 11a) [15], [67]. This increases the deformation resistance and moderates normal stress, thus making the coating less likely to break away from the substrate [68]. Consequently, the nano-mechanical property of the MoS_2_/GO/TiO_2_ coatings is enhanced, as evidenced by a 78.3 % and 9.3 % higher in hardness compared to that of traditional ceramic coating and MoS_2_/TiO_2_ coating, respectively (Fig. 11c). Above all, the enhancement of anti-friction performance in MoS_2_/GO/TiO_2_ coatings does not rely on the continuous release of MoS_2_ and the formation of a lubrication layer in the friction pair to reduce the friction coefficient [14]. Instead, it is driven by the electrochemical process of PEO, regulated by GO, which forms ceramic coatings with MoS_2_ possessing different interlayer spacing. This difference in interlayer spacing induces distinct dislocation evolution and reactions at the incoherent interface formed by MoS_2_ and TiO_2_. On the one hand, the generation of large strain helps release stress, while dislocation reactions facilitate strain release, thereby improving the critical load of the ceramic to resist crack initiation and reduce wear rate. On the other hand, the formation of stable dislocation dipole configuration, in the form of continuous dislocation pinning to enhance the nano-mechanical properties. The dual enhancement of critical load and nano-mechanical properties ensures that the friction coefficient of the MoS_2_/GO/TiO_2_ coating is reduced by 90.0 % compared with that of the traditional ceramic coating, which enhances anti-friction performance and provides a new idea for the preparation of antifriction heterojunction incoherent ceramic coatings.

## 5 Conclusion

In this study, ultrasonic-assisted plasma electrolytic oxidation (PEO) was used to generate MoS_2_/GO/TiO_2_ coatings with varying interlayer spacings. The ultrasonic cavitation effect effectively dispersed GO, enhancing plasma discharge and facilitating the *in-situ* synthesis of MoS_2_ with varying interlayer spacings. The alteration of the interlayer spacing of MoS_2_ also promoted dislocation evolution at incoherent interfaces. At the MoS_2_(0.534 nm)/TiO_2_ interface, dislocation dipoles dissipated residual stress and prevented crack propagation, while at the MoS_2_(0.227 nm)/ TiO_2_ interface, stable dislocation dipoles restricted slip and enhanced mechanical properties. Compared to traditional PEO coatings, this interaction led to a 90.0 % reduction in the friction coefficient and a 91.6 % decrease in wear rate. These results provide valuable insights into dislocation behavior at incoherent interfaces, offering a promising strategy for enhancing the performance of ceramic coatings in high-stress environments. Future researches should focus on optimizing these electrochemical processes and exploring the integration of other two-dimensional materials.

## CRediT authorship contribution statement

**Ziwei Guo:** Writing – original draft, Methodology, Formal analysis, Data curation. **Yongnan Chen:** Writing – original draft, Funding acquisition, Formal analysis, Conceptualization. **Nan Wang:** Writing – review & editing, Visualization, Funding acquisition, Conceptualization. **Yiku Xu:** Writing – review & editing, Supervision, Conceptualization. **Qinyang Zhao:** Supervision, Conceptualization. **Zhimin Hou:** Validation, Formal analysis, Data curation. **Guangrui Gao:** Validation, Resources. **Yan Kang:** Formal analysis, Data curation. **Haifei Zhan:** Validation, Formal analysis.

## Declaration of competing interest

The authors declare that they have no known competing financial interests or personal relationships that could have appeared to influence the work reported in this paper.
